# Time spent in primary care for hip osteoarthritis patients once the diagnosis is set: a prospective observational study

**DOI:** 10.1186/1471-2296-12-48

**Published:** 2011-06-10

**Authors:** Nienke Paans, Willem Jan van der Veen, Klaas van der Meer, Sjoerd K Bulstra, Inge van den Akker-Scheek, Martin Stevens

**Affiliations:** 1 Department of Orthopaedic Surgery, University Medical Center Groningen, University of Groningen, The Netherlands; 2 Department of Orthopaedic Surgery, Martini Hospital Groningen, The Netherlands; 3 Department of General Practice, University Medical Center Groningen, University of Groningen, The Netherlands

## Abstract

**Background:**

Previous research on time to referral to orthopaedic surgery has predominantly used hip complaints as starting point instead of the moment the diagnosis of osteoarthritis (OA) of the hip is established, therefore little is known about the length of time a patient diagnosed with hip OA stays under the care of a general practitioner (GP). No knowledge on factors of influence on this time period is available either. Aim of this study was thus to determine the time an incident hip OA patient stays in the care of a GP until referral to an orthopaedic department. Influencing factors were also analyzed.

**Methods:**

A prospective observational study was conducted based on data over a 10-year period from a general practice-based registration network (17 GPs, > 30,000 patients registered yearly). Patients with the diagnosis of hip OA were included. A survival analysis was used to determine time until referral to an orthopaedic department, and to determine factors of influence on this time.

**Results:**

Of 391 patients diagnosed with hip OA, 121 (31%) were referred; average survival time until referral was 82.0 months (95% CI 76.6-87.5). Less contact with the GP for hip complaints before the diagnosis of hip OA was established resulted in a decreased time to referral.

**Conclusions:**

The results of this study show that patients with hip OA were under the care of a general practitioner, and thus in primary care, for a considerable amount of time once the diagnosis of hip OA was established.

## Background

Hip osteoarthritis (OA) is a common musculoskeletal disorder. In the Netherlands, the number of new cases of symptomatic OA of the hip was found to be 1.7 per 1000 in 2007 [1]. In the US the most recent incidence rates were reported to be 0.9 per 1000 person-years [2]. Patients with end-stage OA often undergo a total hip arthroplasty (THA). THA is a highly successful treatment and the number of people indicated for this treatment is increasing [3,4].

In most European countries general practitioners are the gatekeepers for the medical decision-making [4]. Normally a patient with hip OA stays in the general practice until conservative treatment no longer suffices and hip replacement becomes an option. This is the moment the patients switches from primary to secondary care.

Previous research on the management by GPs predominantly used hip complaints as starting point instead of the moment the diagnosis of hip OA was established [5,6]. Hence little is known about the length of time a hip OA patient generally stays under the care of a general practitioner (GP) from the moment the diagnosis of hip OA is established until referral to orthopaedics - the switch to secondary care. Insight into the length of time from hip OA diagnosis until referral to orthopaedics is of importance because it provides insight into the time available for the application and/or development of non-surgical interventions. This is of major relevance as non-surgical interventions could significantly contribute to postponing the hip replacement which in turn can prevent future revision surgery.

The literature reports variations in management of hip complaints by the GP [5,6] and influencing factors on these variations are suggested, like older age (> 60 years) and a body mass index (BMI) over 30 kg/m^2^[5-7]. The literature lacks information concerning influencing factors for time to referral for patients diagnosed with hip OA specifically though. The aim of the present study was therefore to determine the time an incident hip OA patient stays in the care of a GP until the patient is referred to an orthopaedic department. The presence of influencing factors on the length of this time period was also analyzed.

## Methods

This was an observational cohort study. Data on morbidity and medication were extracted from the Dutch Registration Network Groningen. This general practice-based register was established in 1989, consists of three group practices with about seventeen GPs and is based in the northeastern Netherlands. Participating GPs register all care delivered to their patients. About 30,000 regular patients (24,000 of them aged 18 years or older) are registered yearly. These patients are demonstrated to be representative of the national population.

All consultations, with reasons for the visit as well as diagnoses, referrals and prescriptions, are registered in the RNG. Morbidity data are electronically recorded using the International Classification of Primary Care (ICPC), and each prescribed medication is provided with an ICPC-based indication [8]. This ICPC code is based on a simple biaxial structure consisting of a letter followed by a number. The letter represents a body system (e.g. L = musculoskeletal system), numbers 1-29 provide categories for symptoms and complaints, and numbers 70-99 represent a diagnosis/disease. The medicated prescriptions were automatically classified with an Anatomical Therapeutical Chemical (ATC) code developed by the World Health Organization [9].

The RNG register is a validated [10] and structured register with regular meetings of participating general practices twice a year, for purposes of maintaining an unambiguous registration, which is an ongoing process.

### Patient selection

Incident patients aged 18 or older who received a diagnosis of hip OA (ICPC code L89) in the period between January 1999 and December 2007 were included in this study. The diagnosis of hip OA was based on the definition of OA in the ICPC. The ICPC defines OA of the hip as a joint disorder of at least 3 months duration, with no constitutional symptoms and three or more of the following: intermittent swelling; crepitation; stiffness or limitation of movement; normal ESR, rheumatoid tests, and uric acid; over 40 years of age [8]. Patients with an incidence date preceding their entry to the general practice were excluded from this study (this phenomenon occurs when patients transfer to another general practice and bring along 'historical' data). Patients with time registration errors were also excluded.

### Data selection

**Figure 1:**
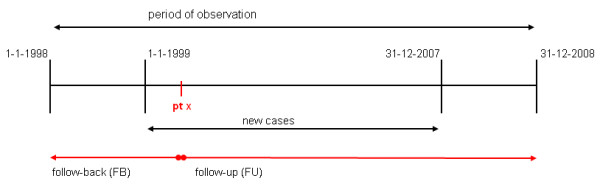
**Timeline of incident cases**.

Registration data from January 1998 to December 2008 were used to obtain a follow-up period (FU) and a follow-back period (FB) of at least one year for all study patients (see Figure 1). The start of the FU period was set at incidence date and the end at occurrence of the event 'first referral to orthopaedics', further addressed as 'referral'. If the event did not take place the end date was set at either the date of censoring or the end of the study period (31-12-2008). A patient was censored when leaving the general practice or in case of death.

The start of the FB period was set at arrival in the general practice or at the beginning of the study period (01-01-1998). The end was set at the last day prior to the patient's incidence date. GP consultations for hip OA (ICPC code L89) in the FU period and hip complaints (ICPC code L13) in the FB period were recorded. These contacts were classified as telephone contact, consultation, house call or medication prescription. Considering that the amount of registered contact or prescriptions is affected by the exposure time during FU or FB, this amount was divided by that specific patient's exposure years (person-years). All GPs were visited to verify exact dates of hip replacements from the electronic surgical reports.

Age at incidence date, sex, different GP practices, number of contact moments during FB, and number of comorbidities and amount of pain medication during FB were assessed as possible influencing factors. Comorbidities were defined as medical conditions such as overweight, obesity, diabetes mellitus, hip fracture, knee osteoarthritis, and cardiac, pulmonary, haematological, renal and oncological diseases. Pain medication was any medication generally prescribed for musculoskeletal pain: prostaglandin synthetase inhibitors, non-steroidal anti-inflammatory drugs (NSAIDs), acetaminophen and opiates (ATC group M01 or N02).

The data used in this study were not publicly available. The patients gave permission for use of their medical data if anonymised. The general practitioners gave permission to provide this anonymized data to the researcher. These GP permissions forms (in Dutch) are available upon request. Finally, the data acquisition was done in accordance with the regulations of the medical ethical board of University Medical Center Groningen.

### Statistical analysis

All statistical analyses were computed using the Statistical Package for the Social Sciences (SPSS, Inc., Version 16.0, 2007, Chicago). Survival was described with Kaplan-Meier survival analyses, which were used to analyze time to referral and the influence of several factors on these survival times. A probability value of less than 0.05 is considered as statistically significant.

## Results

The study group consisted of 391 patients; 72% was female, average age at the incidence date was 66.8 years (SD 14.0) and average exposure in the general practice during the FB period was 4.8 person-years (SD 2.8). Demographics and clinical information of the study group are shown in Table 1.

**Table 1:** Baseline characteristics of study group hip OA patients*

Variables	Hip OA patients (n = 391)
Demographics	
Age at incidence date, years	66.8 (14.0)
Female, %	71.1
Clinical factors	
Exposure during FB, years	4.8 (2.8)
Hip-related contact during FB, person-years, %	
< 1	89.8
1-2	6.4
2 >	3.8
Number of comorbidities during FB, %	
< 2	58.1
2-4	29.2
> 4	12.8

* Values are the mean (SD) unless otherwise indicated.

*OA: osteoarthritis; FB: follow-back*.

**Figure 2:**
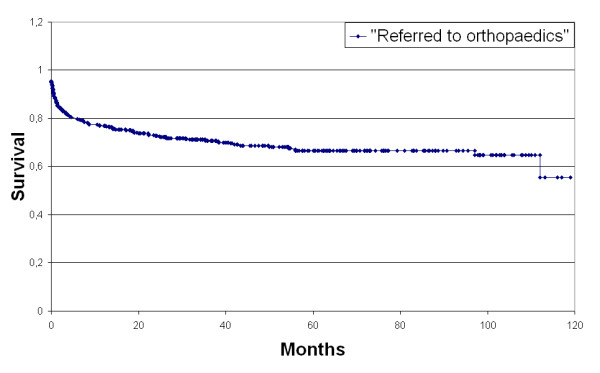
**Survival until referral to orthopaedics**.

Of all patients (N = 391), the average survival time a hip OA patient spent in general practice until referral was 82.0 months (95% CI: 76.6-87.5) (see Figure 2). After a period of 12 months, 90 patients were referred (24%) for the first time, after 36 months 110 patients (37%).

The analysis for factors of influence on survival time showed that 0 to 0.2 GP contact times per year for hip complaints in the FB period significantly decreased the survival time to 78.2 months (95% CI 71.8-84.6). Age, sex, number of comorbidities, amount of pain medication and different GP practices did not influence time to referral (p = 0.925, p = 0.675, p = 0.336, p = 0.223, and p = 0.800 respectively).

## Discussion

This study showed that incident hip OA patients stayed on average 7 years (82 months) under the care of a general practitioner until their referral to orthopaedics. Of all patients in this study, 24% were referred 12 months after hip OA diagnosis and 37% after 36 months. The clinical consequence of this result is the conclusion that a considerable period of time is available to apply non-surgical interventions before surgery is suggested as an option. This is important, as non-surgical interventions could significantly contribute to postponing hip replacement, which in turn can delay or prevent future revision surgery. Future research should therefore explore whether and to what extent non-surgical interventions are applied during this time under the care of a general practitioner.

A previously published American study [11] reported 17.6% referrals to orthopaedics in 20 months. However, that study made a distinction between orthopaedic referrals for evaluation, joint injection or arthroscopy and orthopaedic referrals for actual surgery. Only the latter category was considered as a "referral" for the study's outcome. This difference could explain the lesser number of referrals compared with the present study.

Contrary to earlier research on influencing factors on management of hip complaints, this study showed just one influencing factor on the time until referral to orthopaedics in hip OA: a low frequency of contact for hip complaints with the GP before the diagnosis of hip OA is set accelerates the moment after the GP refers to orthopaedics. In our opinion, the only plausible explanation for this factor is that in patients who postpone GP contact the longest, the OA is so severe and advanced that the GP decides not to delay any further and refers to orthopaedics. However, this cannot be confirmed with our data.

Information about influencing factors on time at the GP practice for patients diagnosed as having hip OA, as presented in this study, is practically never reported in previous studies. Only the previously mentioned American study [11] examined predictors of time to referral to orthopaedic surgery for consideration of joint replacement, and reported that recruitment site was a predictor for time to referral. A possible explanation postulated by the authors for this finding is possible differences in regional waiting lists between the two analyzed groups. In the present study no difference was found in time to referral for the different GP practices. In line with the assumption of this American study, the present finding could be explained by referrals to various orthopaedic departments with separate waiting lists. The present study also presented sex as a non-influencing factor, which was in accordance with the American study [11].

### Strengths and limitations

The present study showed the period of time an incident patient with hip OA currently stays in general practice. To our knowledge, no recent study has reported this information before. It was a strength to have had access to an elaborate prospective database of a medical registration network such as the RNG, enabling us to gather information for an 11-year period spanning from January 1998 to December 2008. In this 11-year period the GPs were not informed about this specific data extraction and there were no limits on their treatment decisions (no fee-for-service). In addition it was a strength that the number of incident patients in the study group and the referral behaviour of the RNG GPs were comparable with national figures: incidence figures of hip OA determined in Dutch GP practices in 2007 [1] were 1.7 per 1000 in one year (versus 1.6 per 1000 in one year in this study). In 2008 GP referral to orthopaedics according to the Netherlands Institute for Health Services Research was 16.4 per 1000 registered male patients and 21.3 for the registered female patient [12]. In the same year the referral behaviour of the RNG network was 16.5 per 1000 patients. A limitation of the study was that only access to information of registered care was gathered, but none about severity of the hip complaints, and no additional investigations were included such as X-rays of the affected hip, as these could have provided more information about the differences between patients at the moment the diagnosis was established.

## Conclusions

Average time to referral was 82 months, i.e. 6.8 years. This indicates that a considerable period of time after the diagnosis is set is spent under the care of a GP, and thus in primary care. The importance of this knowledge can be translated into the assumption that general practice can operate as an optimal setting for the application and/or development of new promising conservative interventions.

## Abbreviations

OA: Osteoarthritis; GP: General Practitioner; THA: Total Hip Arthroplasty; BMI: Body mass index; RNG: Registration Network Groningen; ICPC: International Classification of Primary Care; ATCcode: Anatomical Therapeutical Chemical code; WHO: World Health Organization; FB: follow back; FU follow up; NSAIDs: non-steroidal anti-inflammatory drugs.

## Competing interests

The authors declare that they have no competing interests.

## Authors' contributions

MS, NP, IvdAS and WvdV participated in the design of the study and in collecting the data, performed the statistical analysis and drafted the manuscript. KvdM and SB participated in the progress and revision of the manuscript. All authors read and approved the final manuscript.

## Pre-publication history

The pre-publication history for this paper can be accessed here:

http://www.biomedcentral.com/1471-2296/12/48/prepub
